# Reuse of shipping materials in the intravitreal bevacizumab supply chain: feasibility, cost, and environmental impact

**DOI:** 10.1186/s40942-023-00474-9

**Published:** 2023-06-01

**Authors:** Loi V. Vo, Vanessa Mastrorilli, Alfonse J. Muto, Geoffrey G. Emerson

**Affiliations:** 1 Carl Zeiss Meditec, Inc, Dublin, CA USA; 2 Pine Pharmaceuticals, Tonawanda, NY USA; 3 Retina Consultants of Minnesota, St. Louis Park, MN USA; 4 Retina Consultants of Minnesota, 6099 Wayzata Blvd, Suite #130, 55416 St Louis Park, MN USA

**Keywords:** Intravitreal injections, Sustainability, Greenhouse gas emission, Recycling

## Abstract

**Background:**

Intravitreal injections are the most common ophthalmic procedure worldwide and are also a prime opportunity for waste reduction. This study analyzes the feasibility, environmental impact, and cost of reusing shipping materials for intravitreal injection medications, as compared to wasting coolers and cold packs after single-use.

**Methods:**

In this prospective pilot study, shipping materials (cardboard boxes, polystyrene foam coolers, and cold packs) from repackaged bevacizumab delivered to our clinic (500 doses per week) were saved and reused over a 10-week study period. The shipping supplies were photographed and inspected for defects at point of care (Twin Cities, MN), and returned via standard ground shipping to the outsourcing facility (Tonawanda, NY).

**Results:**

Polystyrene foam coolers (n = 3) survived 10 roundtrips between the outsourcing facility and retina clinic (600 mi each way), although wear-and-tear was visible in the form of marks and dents. Cold packs (n = 35) were less durable, lasting 3.1 ± 2.0 roundtrips. Total carbon dioxide equivalent (CO_2_e) emissions were reduced 43%, by reusing shipping materials (12.88 kgCO_2_e per 1000 bevacizumab doses), as compared to the standard practice of disposing containers after single-use (22.70 kgCO_2_e per 1000 bevacizumab doses), and landfill volume was reduced by 89%. Cost savings from reusing containers offset expenses incurred with return shipping and extra handling in the reuse cohort (net savings: $0.52 per 1000 bevacizumab doses).

**Conclusions:**

Reusing shipping supplies can be cost neutral, with less CO_2_e emissions and reduced landfill. Robust environmental benefit is possible if retina clinics partner with manufacturers to reuse shipping containers.

## Introduction

The healthcare industry consumes a considerable quantity of disposable supplies [1] and is the second largest contributor to landfill in the United States [2] Of the total amount of waste generated by healthcare-related activities, the World Health Organization (WHO) estimates that 85% is general, non-hazardous waste [3]. As waste breaks down, greenhouse gases (GHGs) are emitted and considered causative agents of global warming that drive climate change [4] Of the emitted gases that contribute to global warming, carbon dioxide (CO_2_) is used as the reference gas with total emissions expressed in units called carbon dioxide equivalents (CO_2_eq) [5].

Intravitreal injections are the most common ophthalmic procedure worldwide,^6^ and are also a prime opportunity for waste reduction. Cameron et al. recently reported that shipping waste contributes 83% of the overall landfill produced by intravitreal injection procedures, in the form of “single-use” coolers made from polystyrene foam (e.g. Styrofoam®), cardboard boxes, and disposable cold packs [6]. Foam coolers are of particular concern to the environment because of their large volume in landfill and slow rate of biodegradation, persisting as solid waste for long periods of time [7]. Polystyrene foam cannot be processed, and there are no existing take-back programs within the intravitreal injection market. By contrast, temperature-controlled shipping containers are reused within other branches of healthcare, for example for vaccine distribution [8].

The goal of this study is to demonstrate that foam coolers can be reused by retina clinics for recurring shipments of anti-VEGF, along with cold packs and cardboard boxes. We hypothesized that the reuse of these materials could reduce carbon dioxide equivalent emissions, and further hypothesized that reusing the materials would be financially feasible.

## Materials and methods

Shipping materials related to weekly shipments of repackaged bevacizumab for intravitreal injection (0.05 mL x 500 doses) were collected upon receipt in our clinic (St. Paul, MN) after overnight shipping (United Parcel Service, UPS) from the outsourcing facility (Pine Pharmaceuticals, Tonawanda, NY). The drug was unpacked and stored for use in the clinic. For each shipment, packaging materials consisting of 3 polystyrene coolers, 11 cold packs, and 3 card boxes (all typically discarded) were collected, inspected, and returned to the outsourcing facility via UPS ground shipping. Boxes, coolers, and cold packs were inspected each roundtrip for integrity and reused if undamaged or if only cosmetic changes were visible, such as dents, marks, or scrapes. More substantial defects, such as tears, holes, or structural weakness, triggered replacement of the item. For cold packs, these substantial defects included any breaks in the gel pack plastic cover causing a leak or visible gel through the defect. For the cooler, it included cracks, holes, or openings that might allow passage of air flow. For the box, a tear in the cardboard warranted replacement.

The target temperature for bevacizumab in transit and storage was 2–8 °C (per package insert). Temperature compliance was assessed by reviewing the cold packs inside the cooler to confirm that cold packs were still frozen upon arrival (overnight shipping) from the outsourcing facility, yet the syringes of Avastin were not frozen.

The study period was 10 weeks, consisting of 10 roundtrips for the packing materials. Materials were weighed using a multifunction scale (Greater Goods, B01JTDG084), photographed (iPhone 11), and recorded in a spreadsheet (Microsoft Excel, v. 16.40). The cost analysis was calculated using material and shipping costs available to the outsourcing facility, with applicable bulk discounts. Carbon dioxide equivalent (CO_2_e) was approximated as previously described using estimates from Environmental Protection Agency (EPA) data [9].

This study conforms to the Declaration of Helsinki. The study was deemed exempt from Institutional Review Board (IRB) approval as the collection and analysis of material for this study did not include any human subjects or patient information.

## Results

**Fig. 1:**
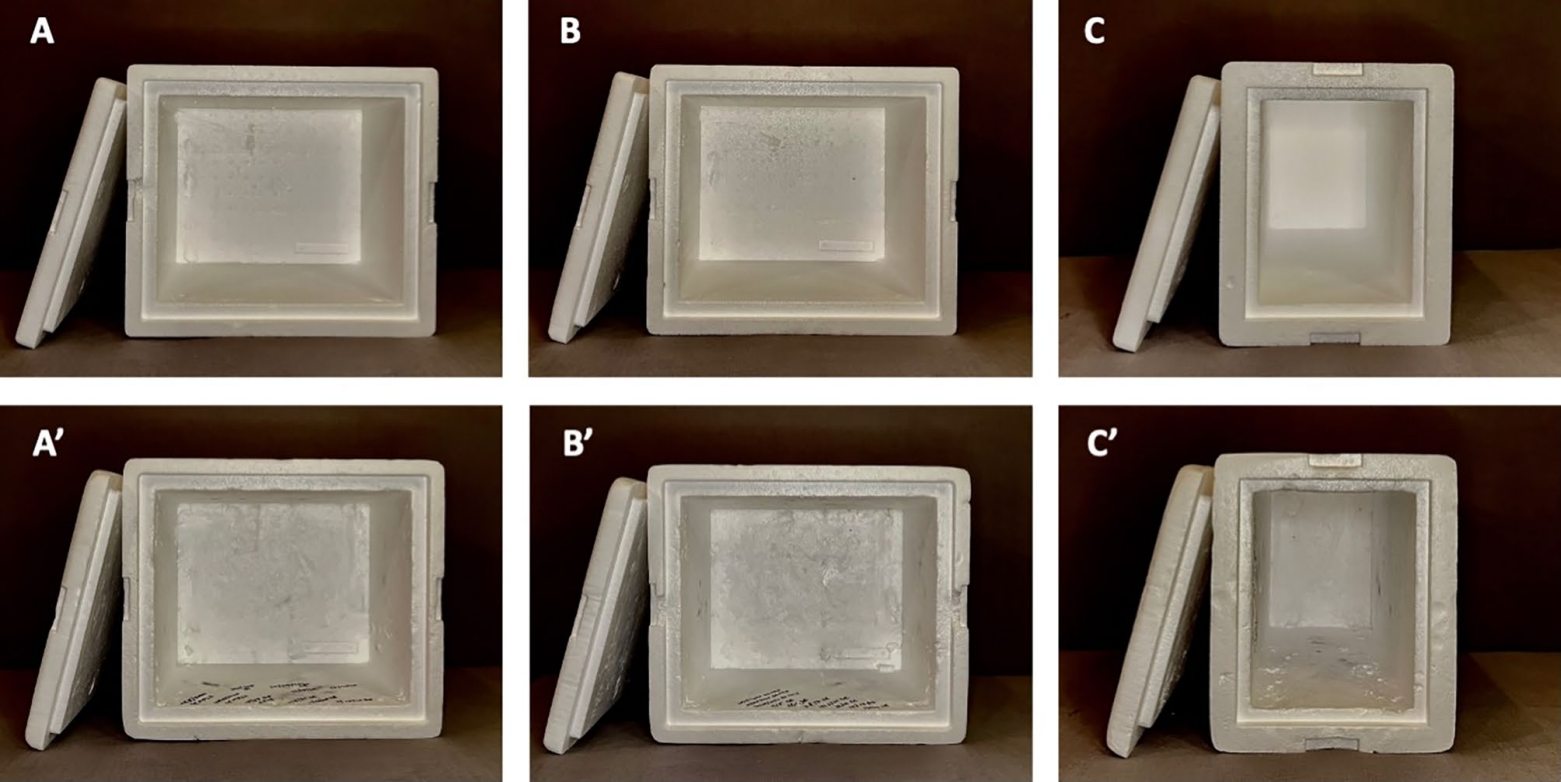
“Single-use” foam coolers before (**A**-**C**) and after (A’-C’) 10 roundtrips between outsourcing facility (Tonawanda, NY) and retina clinic (St. Paul, MN) to transport temperature-controlled doses of bevacizumab. Note that minor wear and tear was visible, in the form of marks and dents in the cooler. However, the contents of the cooler were protected and remained cold throughout the multiple uses

**Fig. 2:**
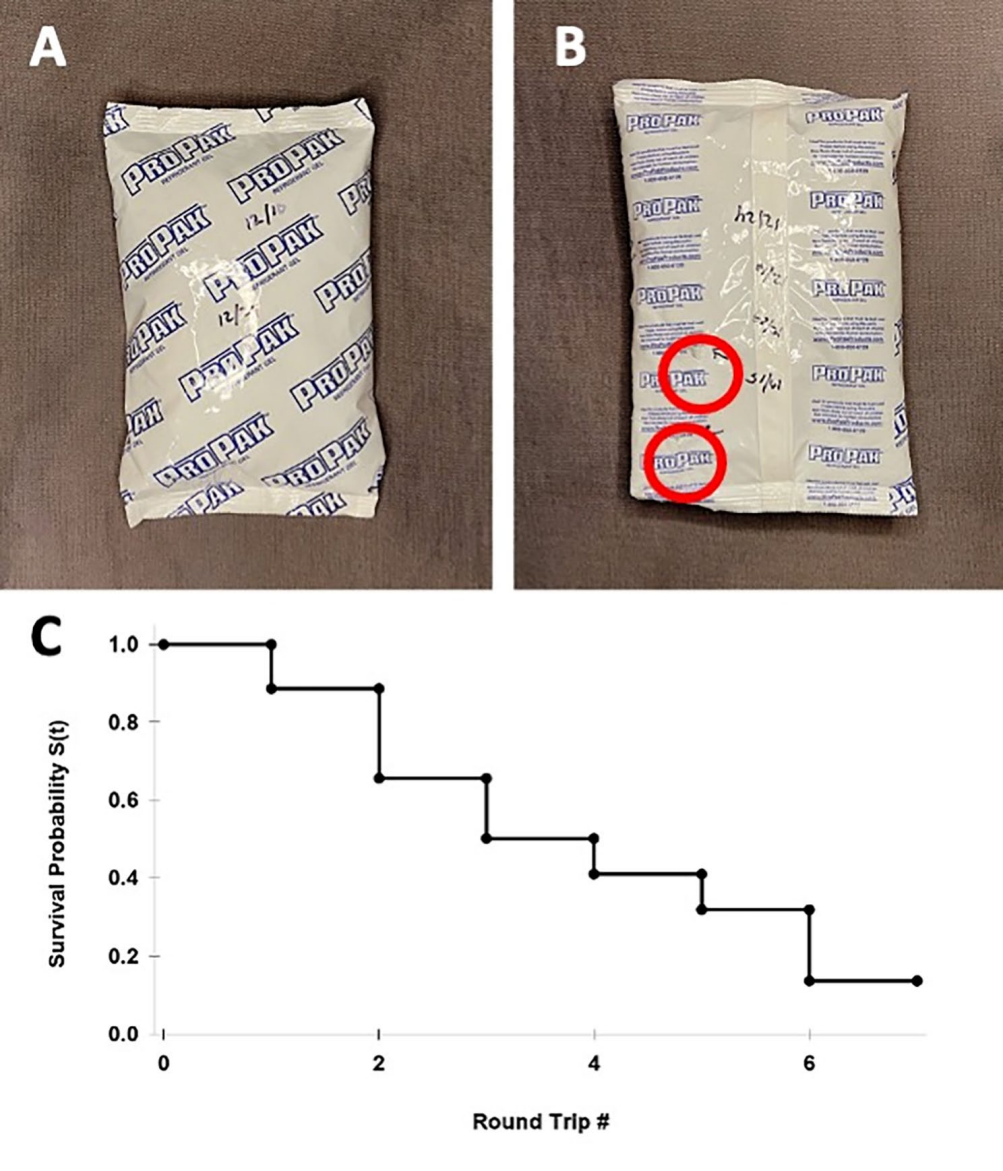
Reused cold pack (**A**) after two shipments, and (**B**) after four shipments, with visible signs of wear and tear (red circles), warranting replacement. Kaplan-Meier plot (**C**) demonstrates the survival probability of a single cold pack versus roundtrip number. Censoring is indicated by black circles

Polystyrene foam coolers (n = 3) survived 10 roundtrips between the outsourcing facility and retina clinic (600 mi each way), maintaining good performance for the duration of the study (i.e., medication stayed cold, and cold packs remained frozen during inbound shipments); however, wear was visible in the form of marks and dents (Fig. 1). Cold packs (n = 35) were less durable, lasting 3.1 ± 2.0 roundtrips (Fig. 2), and cardboard boxes (n = 15) lasted 2 roundtrips only, with visible wear-and-tear already after the first use. Thus, this study demonstrates the feasibility of reusing shipping materials, particularly polystyrene coolers.

Total CO_2_e emissions were reduced 43% by reusing shipping materials, as compared to the standard practice of disposing containers after single use, as shown in Table 1. Landfill volume was reduced by 89% (Table 2). Cost savings from reusing containers offset expenses incurred with return shipping and extra handling in the reuse cohort (net savings: $0.52 per 1000 bevacizumab doses), as shown in Table 3.

**Table 1:** CO_2_e Emissions Related to Shipping of Repackaged Bevacizumab^a^

Component	Standard Practice (kgCO_2_e/1000 doses)	Re-Use (kgCO_2_e/1000 doses)
**Raw Material Extraction & Manufacturing of Shipping Materials** ^**b**^		
Foam (PS)	6.67	0.67
Cardboard	2.54	1.27
Cold Pack Plastic (LDPE)	0.26	0.084
**TOTAL EXTRACTION/MANUFACTURING EMISSIONS**	**9.47**	**2.02**
**Transport of New Shipping Materials to Outsourcing Facility**		
Foam (PS)	0.11	0.01
Cardboard	0.15	0.08
Cold Pack Plastic (LDPE)	0.006	0.0006
**Shipping (Drug + Shipping Materials) from Outsourcing Facility to Clinic**	3.28	3.28
**Return Transport of Used Shipping Materials to Outsourcing Facility** ^**c**^	0	2.68
**TOTAL TRANSPORT EMISSIONS**	**3.54**	**2.77**
**Waste**		
Cardboard Recycling	9.62	4.81
Foam (PS) Landfill^d^	0.053	0.0053
Cold Pack Plastic (LDPE) Landfill^d^	0.003	0.001
**TOTAL WASTE EMISSIONS**	**9.68**	**4.82**
**TOTAL EMISSIONS**	**22.70**	**12.88**

Abbreviations: CO_2_e, carbon dioxide equivalent; PS, polystyrene; LDPE, low-density polyethylene. ^a^ CO_2_e emissions calculated from the US Environmental Protection Agency (EPA) data on greenhouse gas emissions (see References). ^b^ Assumes extraction/manufacturing from virgin materials. ^c^ Assumes ground shipping (600 miles) from clinic to outsourcing facility. ^d^ Emissions solely based on transportation to landfill (20 miles), as PS and LDPE do not contain biodegradable carbon

**Table 2:** Landfill Volume

Component	Standard Practice Volume per 1000 doses (m [3])	Re-Use Volume per 1000 doses (m [3])
Foam (PS)	0.21	0.021
Cold Pack Plastic (LDPE)	0.015	0.0048
**TOTAL VOLUME**	**0.23**	**0.026**

Abbreviations: PS, polystyrene; LDPE, low-density polyethylene

**Table 3:** Shipping and Handling Costs (per 1000 Doses of Repackaged Bevacizumab)

Shipping Package Component	Standard Practice Cost ($) Per 1000 Doses	Re-Use Cost ($) Per 1000 Doses
Shipping Labels	67.59	87.2
Cold Packs	8.25	2.75
Cardboard Boxes	5.8	2.9
Polystyrene Cooler	29.7	2.97
Return Shipping Additional Handling (Labor)^a, b^	-	15
**TOTAL COST**	**$111.34**	**$110.82**

^a^Assumes 15 min of additional handling/labor to turn around reused shipping supplies at clinic, and 15 min additional at outsourcing facility. ^b^Note that return shipping is lower cost because the parcel is lower weight (without cargo), and because the return is via standard ground shipping, as opposed to overnight air shipping

## Discussion

Reusing shipping supplies (foam coolers, cold packs, and cardboard boxes) is feasible for retina clinics, and meaningful in terms of environmental impact. Reuse of these materials saves on CO_2_e emissions, landfill, and cost, compared to wasting shipping materials at point of care after single-use. However, putting circular reuse of shipping materials into practice will take effort and organization; for most busy clinics, “reduce, reuse, recycle” takes a back seat to the exigencies of patient care. Furthermore, take-back programs do not yet exist for any of the anti-VEGF medications, and the success of any such program will depend on the appetite of outsourcing facilities, distributors, and manufacturers to participate.

Assuming 10 million anti-VEGF injections are administered annually in the United States,^11^we estimate that retina practices generate enough “single use” foam coolers to fill six Olympic swimming pools each year. Modest reductions in waste are achievable by buying in bulk, (rather than multiple small orders) and by choosing medication that is packaged more efficiently, such as bevacizumab. The branded drugs have bulkier packaging; thus fewer items fit into a cooler and more shipping materials (cold pack, cardboard, and polystyrene) are necessary per dose [6]. However, the greatest opportunity to reduce landfill is to reuse coolers and cold packs. Recycling of these components is not available, and would still be wasteful because recycling requires additional processing of the materials [10].

A recent carbon footprint analysis of intravitreal injection identified patient travel (77%), procurement (19%) and building energy use (4%) as the largest contributors to carbon emissions [4]. A separate study identified that shipping materials account for 83% of the solid waste produced in the procurement of an intravitreal injection [11]. Because shipping materials are disposed in local landfill rather than incinerated, they account for relatively little of the carbon emissions of a single intravitreal injection (by our estimate, < 2%). Nevertheless, the environmental impact of extruded polystyrene and other packaging materials is more than their kgCO2e due to the relatively large volume in landfill, slow degradation over time, and concern for plastic contaminants in the water supply.

Recent surveys in the United States [12] and Europe [13] show that 93% and 92% of eye surgeons believe there is too much waste in the OR. If there is almost universal agreement, then why do we continue to waste so much? The answer is likely multifactorial [14] but perhaps in part due to a belief among healthcare professionals and regulators that reused materials are unsafe for patient care compared to disposable (new) items. This is not necessarily true, especially for packaging and containers, but without evidence to the contrary, the healthcare system is unlikely to change.

The financial incentive to reuse polystyrene is minimal. The raw materials are inexpensive, and the handling (labor) and return shipping can be costly compared to replacement “single use” containers. Nevertheless, it is important to consider the whole picture, including the repercussions of extracting raw materials, greenhouse gas emissions, and impact of disposal on the environment.

In summary, reusing foam coolers, cold packs, and cardboard boxes is feasible for retina clinics, resulting in less CO_2_e emissions, reduced landfill, and cost savings compared to wasting shipping materials at point of care after single-use. Retina clinics can achieve robust benefits to the environment by partnering with their suppliers to reduce waste.

## Data Availability

The datasets used and/or analyzed during the current study are available from the corresponding author upon reasonable request.

## List of abbreviations

WHO: World Health Organization
GHGs: Greenhouse gases
CO_2_: Carbon dioxide
CO_2_eq: Carbon dioxide equivalents
EPA: Environmental Protection Agency
VEGF: Vascular endothelial growth factor
IRB: Institutional Review Board
